# Mitochondrial Redox Signaling Is Critical to the Normal Functioning of the Neuronal System

**DOI:** 10.3389/fcell.2021.613036

**Published:** 2021-01-28

**Authors:** Olena Odnokoz, Kyle Nakatsuka, Corbin Wright, Jovelyn Castellanos, Vladimir I. Klichko, Doris Kretzschmar, William C. Orr, Svetlana N. Radyuk

**Affiliations:** ^1^Department of Biological Sciences, Southern Methodist University, Dallas, TX, United States; ^2^Oregon Institute of Occupational Health Sciences, Oregon Health and Science University, Portland, OR, United States

**Keywords:** mitochondria, redox state, peroxiredoxin, neuronal function, aging, *Drosophila*

## Abstract

Mitochondrial dysfunction often leads to neurodegeneration and is considered one of the main causes of neurological disorders, such as Parkinson's disease (PD), amyotrophic lateral sclerosis (ALS) and other age-related diseases. Mitochondrial dysfunction is tightly linked to oxidative stress and accumulating evidence suggests the association between oxidative stress and neurological disorders. However, there is insufficient knowledge about the role of pro-oxidative shift in cellular redox and impairment of redox-sensitive signaling in the development of neurodegenerative pathological conditions. To gain a more complete understanding of the relationship between mitochondria, redox status, and neurodegenerative disorders, we investigated the effect of mitochondrial thiol-dependent peroxidases, peroxiredoxins (Prxs), on the physiological characteristics of flies, which change with pathologies such as PD, ALS and during aging. We previously found that through their ability to sense changes in redox and regulate redox-sensitive signaling, Prxs play a critical role in maintaining global thiol homeostasis, preventing age-related apoptosis and chronic activation of the immune response. We also found that the phenotype of flies under-expressing Prxs in mitochondria shares many characteristics with the phenotype of *Drosophila* models of neurological disorders such as ALS, including impaired locomotor activity and compromised redox balance. Here, we expanded the study and found that under-expression of mitochondrial Prxs leads to behavioral changes associated with neural function, including locomotor ability, sleep-wake behavior, and temperature-sensitive paralysis. We also found that under-expression of mitochondrial Prxs with a motor-neuron-specific driver, D42-GAL4, was a determining factor in the development of the phenotype of shortened lifespan and impaired motor activity in flies. The results of the study suggest a causal link between mitochondrial Prx activity and the development of neurological disorders and pre-mature aging.

## Introduction

The mitochondrion is an organelle that plays a key role in the control of many cellular processes. Given the ability of mitochondria to act as the primary generator of reactive oxygen species (ROS), maintaining a balanced redox status in this organelle is of paramount importance for normal cell function. It is known that mitochondria and redox signaling play an important role in maintaining normal functioning of different organs and tissues in a wide range of species (Amigo et al., 2016). Studies in model organisms have shown that dysregulation of signaling pathways in a single tissue can significantly affect organismal longevity (Grotewiel et al., 2005). Altered or dysregulated mitochondrial redox state and increased oxidative stress (OS) underlies pre-mature aging and many pathological conditions, including neurodegenerative disorders such as Parkinson's disease (PD) (Xia et al., 2020) and amyotrophic lateral sclerosis (ALS) (Wang et al., 2018).

The redox state in mitochondria is maintained by many factors, among which are thiol-dependent peroxidases or peroxiredoxins (Prxs). Prxs are able to sense and regulate cellular concentrations of hydrogen peroxide and other peroxides, thereby acting as antioxidants and regulators of cellular redox and redox-sensitive signaling pathways. Prxs are found in virtually all phyla (Rhee et al., 1999), and their representatives are documented in invertebrates, including *Drosophila*, which possess all of the Prx mammalian homologs. There are two subtypes of Prxs in mitochondria, Prx3 and Prx5. These Prxs are implicated in the development of various neurological disorders, as shown experimentally using transgenics and mutants (Chen et al., 2012, 2014; Davey and Bolanos, 2013; Angeles et al., 2014; Kim et al., 2016; Park et al., 2017; Agrawal and Fox, 2019; Pharaoh et al., 2019; Wang et al., 2019; Lee et al., 2020).

We have previously investigated the functions of mitochondria-localized Prxs (Prx3 and Prx5) in *Drosophila* and found broad effects on the redox environment, tissue-specific apoptosis, life span, resistance to OS, geotaxis, and the immune response (Klichko et al., 2019). The most dramatic effects were observed in flies called double mutants (DM) that under-express both Prxs (Radyuk et al., 2010). We also found that these changes in cellular function and physiology in the DM were largely similar to those seen in normal physiological aging, but at an accelerated pace, suggesting that these Prxs interact with longevity pathways.

Here, we have expanded our research to investigate the potential links between mitochondrial Prxs and neuronal function and to determine the role of mitochondrial Prxs in the development and progression of neurodegenerative disorders. It is established that OS and impaired redox status, as well as dysfunctional mitochondria, correlate with neuroinflammation and the development of many age-related neurodegenerative disorders (De Rose et al., 2017; Kumar et al., 2017; Xia et al., 2020). However, while the connection between OS and inflammation is established, the mechanistic underpinnings have not been sufficiently delineated.

We set two goals: (a) to further characterize Prx mutants, focusing on behavioral characteristics associated with neural function; (b) to identify the critical tissues responsible for the DM phenotype.

## Materials and Methods

### Fly Strains and Procedures

All mutant, transgenic, and enhancer fly lines were backcrossed into the *y w* reference strain background a minimum of 8 times. The *daughterless* Da-GAL4, Appl-GAL4, and D42-GAL4 driver lines were supplied by Dr. Blanka Rogina (University of Connecticut Health Science Center). Properties of the drivers are described in FlyBase and in publications (Parkes et al., 1998; Taghert et al., 2001; Orr et al., 2005; Legan et al., 2008). The *dprx5* mutant allele is described in Michalak et al. (2008). Under-expression of dPrx3 was achieved using UAS-RNAi-*dprx3* transgenic fly lines described in Radyuk et al. (2010). Under-expression of dPrx3 by RNAi globally and in neuronal tissues was achieved by crossing the UAS-RNAi*-dprx3* transgene to Da-GAL4 or Appl-GAL4 and D42-GAL4 drivers correspondingly. The genotypes of the flies and abbreviations are shown in Table 1.

**Table 1:** Genotypes of control and experimental flies and fly line names.

**Line name**	**Genotype**
*dprx5*	*dprx5*/ Da-GAL4, *dprx5*
*dprx3*	RNAi-*dprx3*/ Da-GAL4, *dprx5*
DM	RNAi-*dprx3, dprx5*/ Da-GAL4, *dprx5*
Control	+/ Da-GAL4, *dprx5*
Appl DM	Appl-GAL4/ +; RNAi-*dprx3, dprx5*/ *dprx5*
Appl Control	Appl-GAL4/ +; *dprx5*/ +
D42 DM	D42-GAL4/ +; RNAi-*dprx3, dprx5*/ *dprx5*
D42 Control	D42-GAL4/ +; *dprx5*/+

*DM – flies underexpressing dPrx3 with Da-GAL4 driver in dprx5 mutant background; Appl DM – flies underexpressing dPrx3 with Appl-GAL4 driver in dprx5 mutant background; D42 DM – flies underexpressing dPrx3 with D42-GAL4 driver in dprx5 mutant background. Controls were heterozygous flies carrying driver and transgene alleles. Since there was no significant difference in effects on lifespan and other fly characteristics between driver and transgene controls (17), we only present here the data for one of them, +/Da-GAL4, dprx5 obtained in the current experiments. Appl Control and D42 Control–corresponding driver controls for fly lines under-expressing Prxs with these drivers*.

In all experimental studies, flies were cultured on standard sucrose-cornmeal fly food at 25°C. Age-synchronized cohorts of flies were generated by collecting newly-enclosed flies over a period of 48 h. Approximately 25 flies were placed in each vial and transferred to fresh food on a daily basis. Survivorship studies were conducted as described in our previous publications (Radyuk et al., 2010; Odnokoz et al., 2016). Fly deaths were recorded approximately every 24 h.

### Negative Geotaxis Assay

The negative geotaxis (climbing assay) was performed according to Pendleton et al. (2002) and Ali et al. (2011) with some modifications. Briefly, flies were placed in an empty glass vial. After 10 min of acclimation the flies were gently tapped down to the bottom of the vial and allowed to climb for 30 s. A number of flies that are able to climb or jump ~4 cm distance and to reach the top of a vial was counted. The assay was repeated for the same group in triplicate, allowing for 10 min rest period between each trial. The geotaxis was expressed as a number of climbers/jumpers to the total number of flies. Studies were performed at 25°C under standard lighting conditions.

### Phototaxis Assay

Phototaxis was evaluated essentially as described in Vang et al. (2012). The vial containing 25 flies was left for 30 min in the dark room to allow adaptation of the flies to darkness. Dim yellow illumination was turned on so the flies could be seen in absence of the white light. The vial then was attached to the 20 cm test tube and place horizontal and perpendicular to the light source 15 cm away. The light source was turned on and number of flies was counted after 2 min in the last quarter of the apparatus.

### Sleep-Wake Behavior

TriKinetics Locomotor Activity Monitoring System (TriKinetics Inc.), Data Acquisition (DAMSystem308X) and File Scan (DAMFileScan110X) Software were used to measure sleep-wake behavior. Studies were done under a 24 h light/dark cycle regimen (12 h:12 h LD) at 25°C, 50% humidity. For single fly sleep-wake behavior study, we used Trikinetic Activity Monitor 1 (http://www.trikinetics.com/). Flies were placed into separate 10 × 0.5 cm tubes with fly food from one side and closed with cotton from other side. As a fly walks from end to end back and forth, its passage is detected and counted by an infrared beam, which is located in the middle of the tube.

Single fly activity data was analyzed using R version 3.1.2, RStudio Version 0.98.1091, Microsoft Excel Version 14.6.1, and Prism 5.0c (GraphPad Software, CA) to calculate total activity, total sleep and night activity over time. R script was written and used to organize the data in the format represented in **Figures 3**, **4** to calculate the various parameters (available upon request). Flies were transferred to clean tubes with fresh food every 2–3 days. Day of fly death was identified from activity measurements. Data was recorded as number of crossings per 5-min bin. Sleep was counted if the fly was inactive for a 5-min period (Shaw et al., 2000). Night activity offset, an index of circadian rhythmicity, was measured as a time between lights off and the proceeding end of an activity bout.

### Acquired Temperature-Sensitive Paralysis

The assay developed by Dr. Rogina's research group (30 2538) has been largely adapted to assess neuronal function. Since we were unable to accurately replicate the analysis because very few flies became paralyzed when held at 30–40°C, presumably due to differences in the genetic backgrounds, a modified version of the procedure has been conducted using the following approach. Flies were collected at several time points (young, middle, and old) predicted to match percentage of lifespan in control flies and short-lived mutants. Flies were exposed to 45.5°C for 30 s, which led to paralysis in 100% of flies at all ages. Then the time that it took for 50% of the flies in each trial to recover from paralysis was measured. This modified approach allowed for more precise and replicable measurements. This modified method demonstrates the same scaling with age across multiple genotypes as reported by Reenan and Rogina (2008), and is coherent with the age-associated decline in neuronal excitability hypothesized to underlie the temperature-sensitive paralysis phenotype.

### Statistical Methods

Statistical analysis was performed using GraphPad Prism 5.0c and Microsoft Excel. The mean survivorship time and statistical significance of differences between survival curves were assessed using the log rank test. Differences in the behavior activity levels were compared between groups by analysis of variance. For multiple comparisons, Bonferroni correction has been used. Statistical significance of the age-specific variations in the activity levels between fly lines was determined by comparing the slopes and intercepts among regression lines. Differences were considered statistically significant at *p* < 0.05. Sample size and statistical methods are listed in details in Figure and Table Legends.

## Results

### Life Span Was Shortened in Flies Underexpressing Mitochondrial Prxs in Neuronal Tissues

It has previously been noted that under-expression of mitochondrial Prxs is particularly damaging to certain tissues, leading to apoptosis in the cardia, intestinal epithelium, oenocytes and thoracic muscles, while no significant pro-apoptotic changes were found in the brain (Radyuk et al., 2009, 2010). Similar characteristics have also been reported in normal flies during aging (Zheng et al., 2005). These observations suggest that changes in mitochondrial Prx levels in certain tissues or cells may be particularly important in the development of the “rapid death” phenotype of the double mutant.

To uncover the potential Achilles' heel responsible for the aberrant neurological behavior of the DM, we investigated the tissue-specific effects of dPrx under-expression using pan-neuronal (Appl-GAL4) and motor neuron-specific (D42-GAL4) drivers. To achieve under-expression of both mitochondrial Prxs together, we targeted the expression of UAS-RNAi-*dprx3* transgene to motor neurons using the D42-GAL4 driver and also to a broad range of the brain neurons using the Appl-GAL4 pan-neuronal driver in a *prx5* null background.

**Figure 1:**
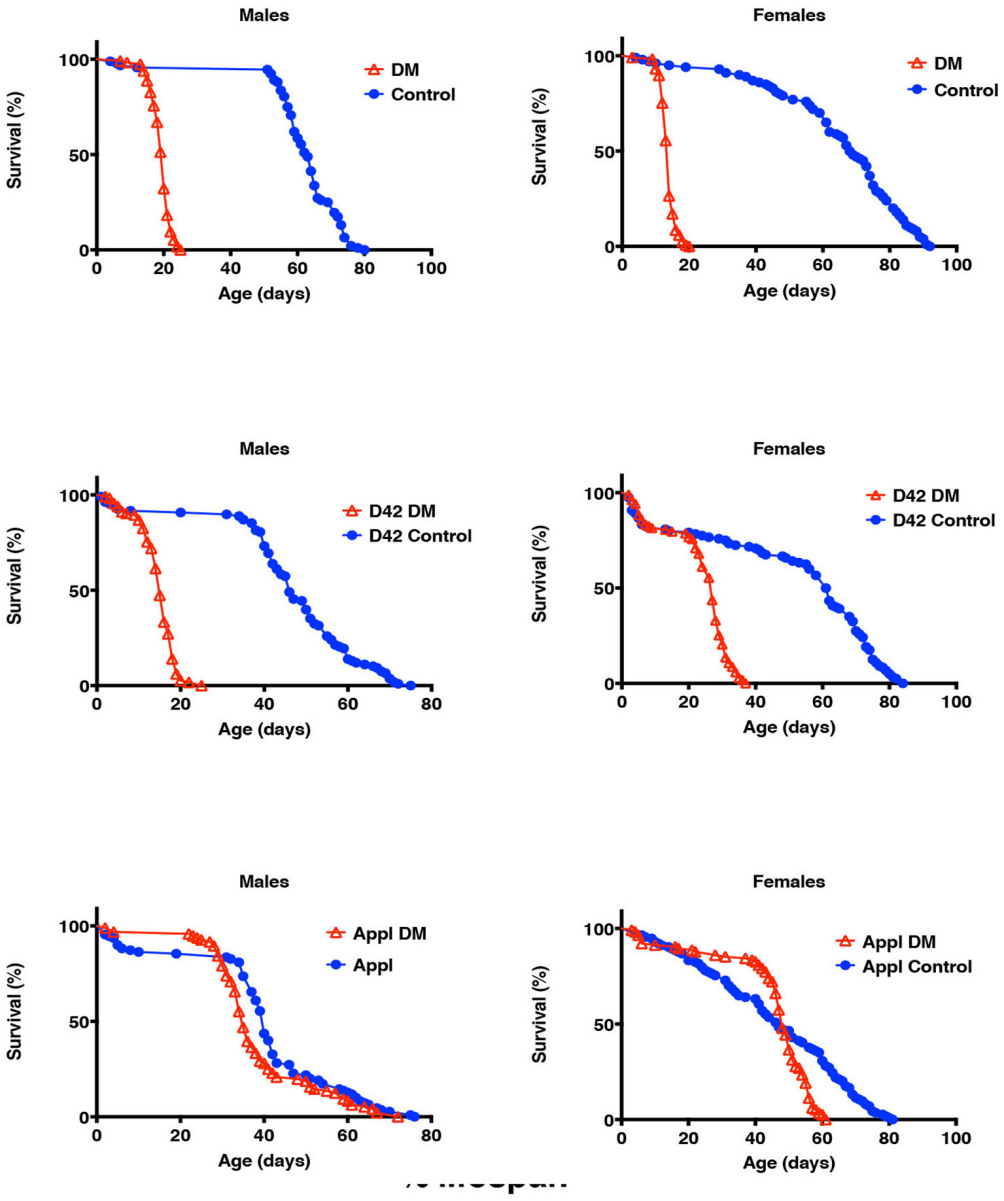
Effects of under-expression of mitochondrial Prxs on fly life spans. dPrx3 was under-expressed in *dprx5* null background globally with Da-GAL4 driver (DM), pan-neuronally with Appl-GAL4 driver (Appl DM) and in motor neurons with D42-GAL4 driver (D42 DM). Approximately 100–125 flies were used for each fly line. Shown are representative data of two independent biological experiments. Similar results were obtained in the biological replicate experiment. The data are summarized in Table 2. The names of fly lines and genotypes of flies are described in Table 1.

The under-expression of mitochondrial Prxs with Appl-GAL4 pan-neuronal driver showed very little to no effect on longevity. In contrast, the under-expression of dPrx3 specifically in the motor neurons in a *prx5* null background had dramatic effects on longevity in both males and females (Figure 1, Table 2). The shortening of life span in the motor neuron-specific DM flies was comparable to that in the flies under-expressing mitochondrial Prxs globally, suggesting that the mitochondrial Prxs in motor neurons are critical for survivorship and maintenance of normal life span.

**Table 2:** Mean life span of the double-mutant flies under-expressing dPrx3 in *dprx5* null mutant background shown in Figure 1.

**Males**				**Females**			
**Line**	**Mean, days 1**	**% vs. control 2**	***p*-value 3**	**Line**	**Mean, days 4**	**% vs. control 5**	***p*-value 6**
Da Control	63 63			Da Control	69 72		
DM	20 19	**31.7 30.2**	**<0.0001 <0.0001**	DM	14 15	**20.3 20.8**	** <0.0001 <0.0001**
APPL Control	36 40			APPL Control	47 35		
APPL DM	36 35	0.0 **87.5**	0.5035 **0.0112**	APPL DM	48 46	102.1 **76.1**	0.1293 **0.0002**
D42 Control	46 44			D42 Control	61 51		
D42 DM	15 14	**32.6 31.8**	** <0.0001 <0.0001**	D42 DM	27 25	**43.9 48.0**	** <0.0001 <0.0001**

*Columns 1 and 4 indicate mean life spans of two independent biological experiments; columns 2 and 5 indicate the percent changes in experimental groups vs. corresponding controls; columns 3 and 6 indicate the significance probabilities of comparisons of survival curves obtained by the log rank tests. Statistically significant differences are shown in bold*.

### The Effects on Behavior: Geotaxis and Phototaxis

Sleep-wake behavior, climbing behavior (negative geotaxis) and reaction to a light stimulus (phototaxis) are parameters commonly used to study the age-related changes in reflex locomotor behavior in *Drosophila* (Gargano et al., 2005).

**Figure 2:**
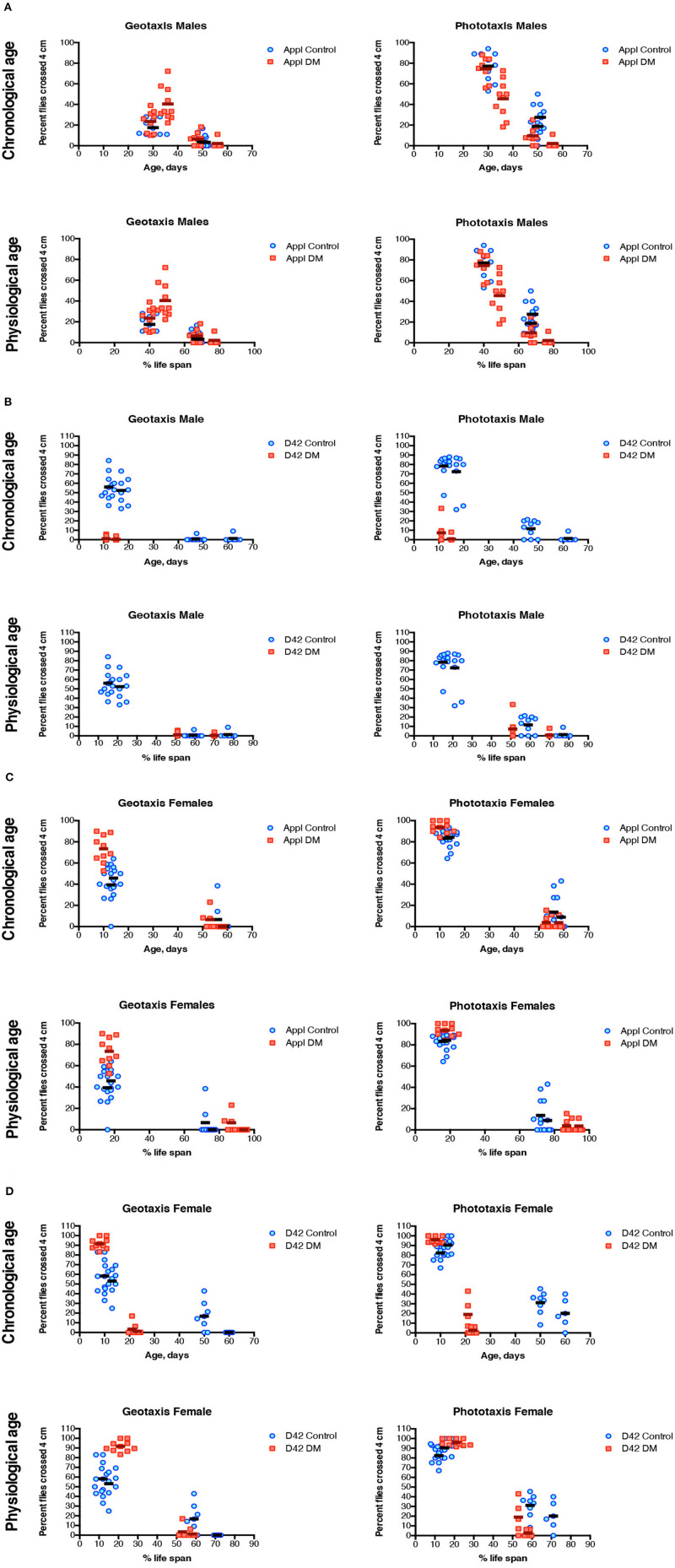
Negative geotaxis and phototaxis of DM flies under-expressing dPrx3 pan-neuronally **(A,C)** and in motor neurons **(B,D)**. The measurements were performed at different ages as indicated in Supplementary Table 1. Results are represented for each single measurement of two experiments with independent cohorts with mean values. The abbreviations and genotypes of flies are described in Table 1. To evaluate the differences in age-dependent changes in sleep-wake behavior parameters between controls and the DM fly lines regression curve slopes and intercepts were compared (Table 3).

Negative geotaxis and phototaxis were measured in both sexes in the motor neuronal double mutant (D42 DM) with corresponding driver control (D42 Control) and in the pan-neuronal double mutant (APPL DM) with corresponding driver control (APPL Control) at multiple time points as indicated (Supplementary Table 1). Since differences in lifespans that lasted for a shorter period in D42 DM, the data were also plotted as a function of physiological age. Both Control and the DM flies showed age-dependent decline in the ability to climb and to walk toward the light source (Figure 2).

There was little difference in negative geotaxis and phototaxis between flies underexpressing Prxs with APPL driver and their corresponding APPL driver control. Control and mutant flies showed similar age-dependent decreases in both sexes (Figures 2A,C).

In contrast, both males and females underexpressing Prxs with the D42 driver showed a steep decrease in negative geotaxis and phototaxis in 10 day-old D42 DM flies relative to the D42 Control (Figures 2B,D). Analysis of the trajectories of these changes during physiological aging also showed significant differences in the slopes (Table 3) due to the greater ability of younger flies to climb and to walk toward a light source.

**Table 3:** Statistical analysis of geo- and phototaxis scaled to percent life span, relied on comparison of the regression curve slopes and intercepts.

**Parameters**	**Slope, *p*-value**	**Intercepts, *p*-value**
**Males, Appl Control vs. Appl DM**		
Geotaxis	0.1433	**0.0008**
Phototaxis	0.8598	**0.0158**
Males, D42 Control vs. D42 DM*		
Geotaxis	0.4057	0.7682
Phototaxis	0.3775	**0.0232**
**Females, Appl Control vs. Appl DM**		
Geotaxis	**0.0095**	
Phototaxis	0.4921	**0.0001**
**Females, D42 Control vs. D42 DM**		
Geotaxis	**<0.0001**	
Phototaxis	**<0.0001**	

*Statistical analysis of age-dependent changes in negative geo- and phototaxis between Appl DM, D42 DM and their corresponding controls. To compare the difference of trajectories of these changes during physiological aging, comparison of regression curve slopes and intercepts was performed using Prism GraphPad Software. Statistically significant differences are shown in bold*.

There were no significant differences in phototaxis and negative geotaxis between the D42 DM and Control when scaled to physiological age (approximately at 60% of their respective life span, which corresponded to ~10 and ~50 days of chronological age of D42 DM and D42 Control, respectively – Supplementary Table 1). Thus, the depletion of mitochondrial Prxs in motor neurons accelerates the decline in locomotor behavior, and this decline follows similar changes as control when scaled to corresponding % of lifespan, or physiological age.

### Age-Dependent Changes in Sleep-Wake Behavior in Prx Mutants

During aging, flies, like other organisms, including humans, experience changes in behavioral characteristics. In particular, characteristics associated with neural function, such as locomotion and circadian rhythm, gradually decrease while sleep fragmentation and sleep duration increase (Jones and Grotewiel, 2011; Koudounas et al., 2012).

Previously, we have found that underexpression of mitochondrial Prxs results in changes in biochemical and physiological characteristics, similar to those observed in flies during aging (Klichko et al., 2019). Here, we expanded the study and examined the effects of underexpression of mitochondrial Prxs on locomotor activity and sleep-wake behavior as an indicator of neuronal health in flies of different ages. Locomotor activity in the Prxs mutants and control flies was continuously monitored across their entire life span by using the locomotor activity monitoring devices from Trikinetics Inc (see Material and Methods).

To investigate changes scaled to the corresponding life spans, flies were collected at different chronological, but at equivalent physiological ages denoted as % of life span (Supplementary Table 2).

**Figure 3:**
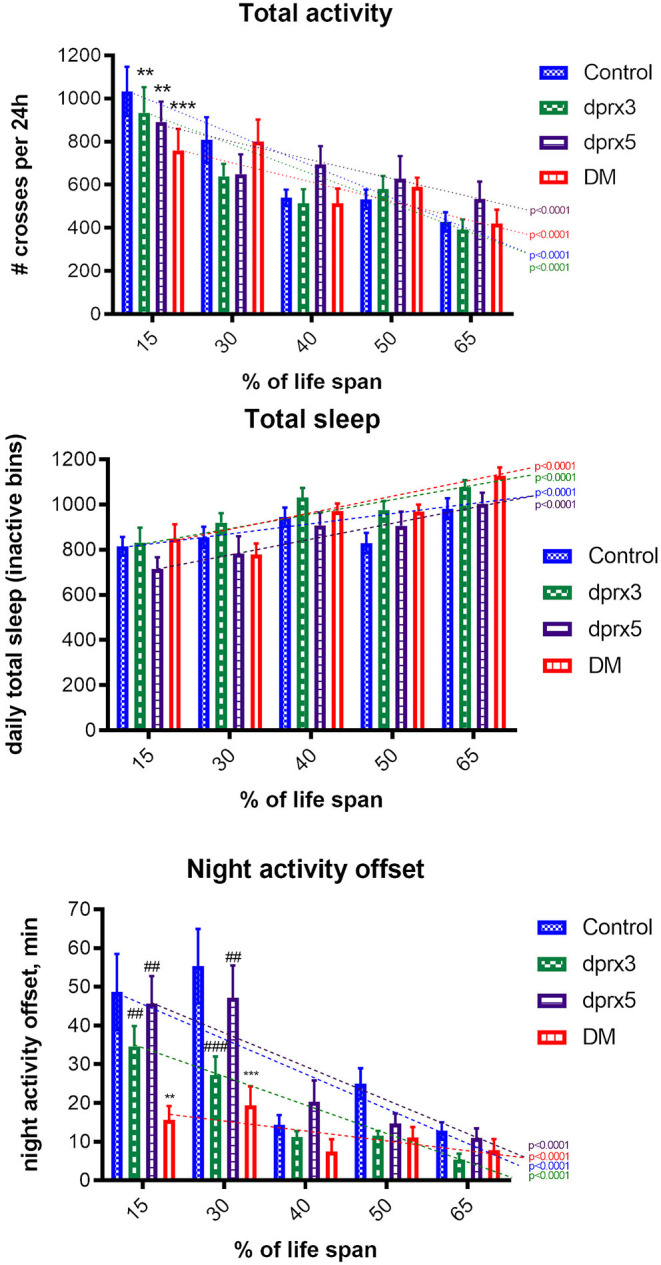
Sleep-wake behavior of single fly. Analysis of daily total activity, daily total sleep and night activity offset in control (Control), single (*prx3* and *prx5*) and Prx double mutants was undertaken in male flies at different physiological ages (Supplementary Table 2). All measurements were performed from Zeitgeber time 0 (ZT0) to ZT24. Results are means ± SEM (*n* = 14–16) for each time point of control and single mutants. The dotted lines represent trend lines of changes during aging, and *P*-values are shown for each fly line. There was a significant decrease in 24-h total activity during aging in all four fly lines (*p* < 0.0001). Statistical analysis has been done by two-way ANOVA with Bonferroni post-tests: Control vs. *dprx3*, Control vs. *dprx5* at 15% life span (*p* < 0.01); Control vs. DM at 15% life span (*p* < 0.001). No statistically significant difference in the changes during aging was found between Control, the double and single mutant lines. The Bonferroni post-test showed a significant difference only between the mean values at 15% of life span. There was a gradual increase in 24-h total sleep during aging in all four lines resulting in statistically significant differences between flies at 15 and 65% of their life spans (*p* < 0.0001). There was no statistically significant difference in the changes during aging between Control, the double (DM) and single mutant lines. There was a significant decrease in night activity offset during aging in all four lines (*p* < 0.0001). Bonferroni post-tests: Control vs. *dprx3* at 30% life span (*p* < 0.001); control vs. DM at 15% (*p* < 0.01) and 30% life span (*p* < 0.001), *dprx3* vs. DM at 15% life span (*p* < 0.01), *dprx5* vs. DM at 15 and 30% life span (*p* < 0.01). To compare the differences between different ages in control and mutant lines, we used two-way ANOVA analysis. To compare differences between means at each % of life span, we used Bonferroni post-tests, Prism software. *compare to control (*p < 0.05, **p < 0.01, ***p < 0.001); ^#^compare to DM (#*p* < 0.05, ##*p* < 0.01, ###*p* < 0.001). *P* values marked by “*” indicate difference between mutants and control. *P* values marked by “^#^” indicate difference between single mutants and DM.

The study of the activity patterns during a daily light-dark cycle showed similar age-dependent changes in the double and single mutants and control when they were normalized to percentage of life span (Figure 3). The total activity, which represents the number of beam crosses over 24 h significantly decreases with age (Figure 3). In contrast, the total sleep or inactivity of flies significantly increased with age (Figure 3). The night activity offset, which represents how long the fly was active after lights go off (ZT12), was significantly decreased during aging and the decrease was more prominent in the DM (Figure 3). Thus, in the double mutant flies with depletion of both Prx3 and Prx5, changes in sleep-wake behavior were similar to those during normal aging, but occurred over a shorter period of time. Moreover, in contrast to control and single mutants, the DM already exhibited a reduction in night activity in chronologically and physiologically young flies.

**Figure 4:**
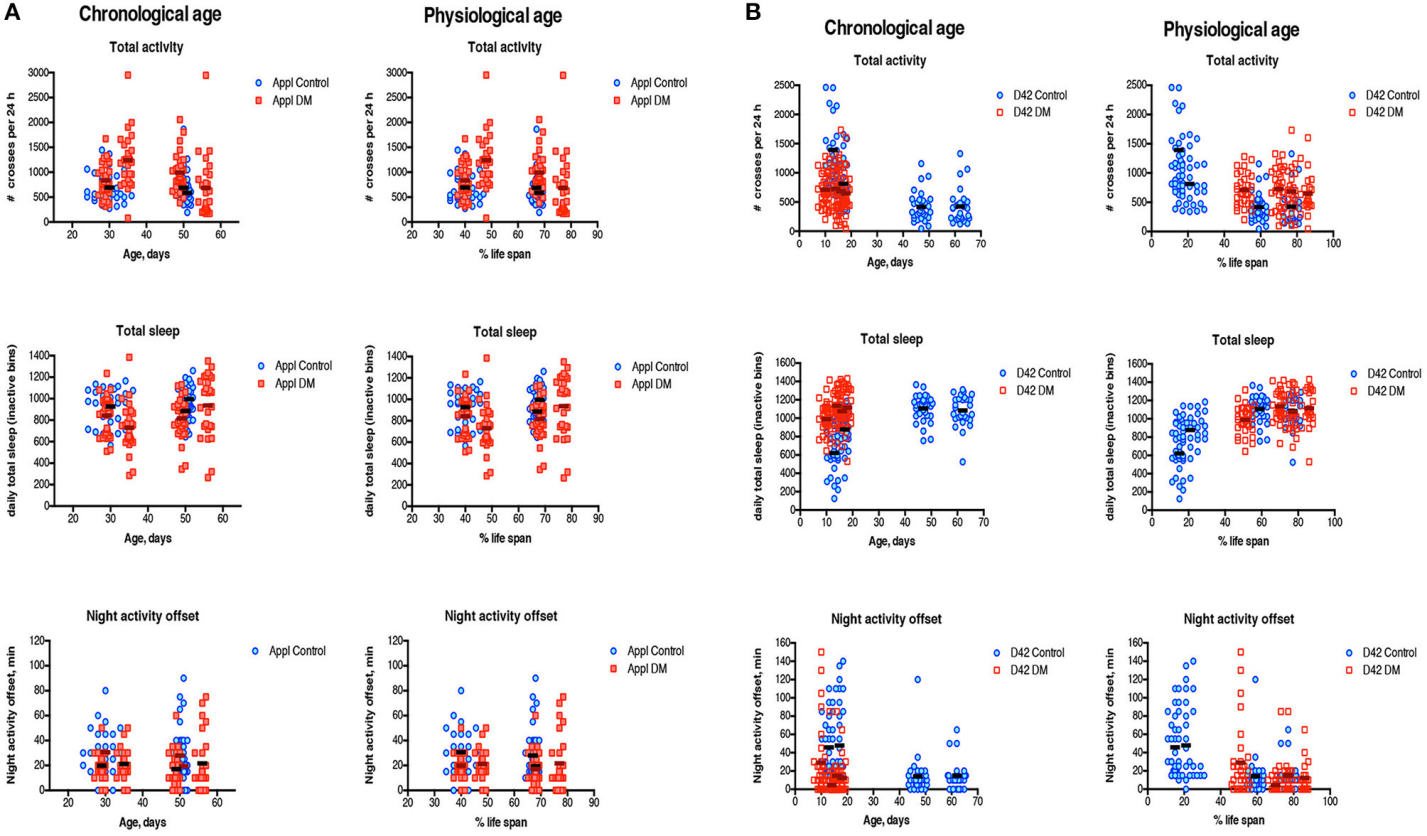
Sleep-wake behavior of single fly. Measures of daily total activity, daily total sleep and night activity offset in the DM obtained with Appl-Gal4 driver **(A)** and D42-Gal4 driver **(B)** and corresponding Controls have been done in male flies at different ages as indicated in Supplementary Table 3. Data are scaled to both chronological age and physiological age. All measurements were performed at Zeitgeber time 0 (ZT0), the time when the lights go “on.” Results are represented for each single measurement of two experiments with independent cohorts with mean values. The abbreviations and genotype of flies are described in Table 1. To evaluate the difference in age-dependent changes in sleep-wake behavior parameters between Control and DM lines, regression curve slopes and intercepts were compared (Table 4).

We also measured different sleep-wake parameters, including daily total activity, total sleep intervals and night activity offset, all known to change with age (Koudounas et al., 2012), in flies under-expressing Prxs pan-neuronally and in motor neurons. There were no significant differences between APPL DM and control at either chronological or physiological ages (Figure 4A, Table 4). In both control and APPL DM mutant, activity slightly declined during aging while sleep duration increased. The data indicate that in APPL mutant sleep-wake behavior was not affected by Prx underexpression.

**Table 4:** Statistical analysis of sleep-wake behavior parameters scaled to percent of life span, relied on comparison of regression curve slopes and intercepts.

**Sleep-wake parameters**	**Appl Control vs. Appl DM**		D42 Control vs. D42 DM*	
	**Slope, *p*-value**	**Intercepts, *p*-value**	**Slope, *p*-value**	**Intercepts, *p*-value**
Daily total activity	0.4232	**<0.0001**	0.7117	**<0.0001**
Daily total sleep	0.2635	**0.0004**	0.0910	0.3542
Night activity offset	0.1773	**0.0057**	0.1415	0.6584

*Statistical analysis of age-dependent changes in sleep-wake behavior parameters between DM and corresponding controls depicted in Figure 4. To compare the difference of trajectories of these changes during physiological aging, we performed comparison of regression curve slopes and intercepts using Prism GraphPad Software. Statistically significant differences are shown in bold*.

In flies under-expressing Prxs in motor neurons, sleep patterns were age-dependent (Figure 4B). The pattern of changes in all sleep-wake parameters was similar in the D42 DM and Control flies, starting at 45% of life span when scaled to lifespan (Figure 4B, Table 4). There were no significant differences between 10 da old D42 DM and 10 da old Control flies (Figure 4B), suggesting that function of motor neurons affected in D42 DM does not influence sleep-wake behavior.

### Age-Dependent Changes in Acquired Temperature-Sensitive Paralysis in the Double and Single Prx Mutants

**Figure 5:**
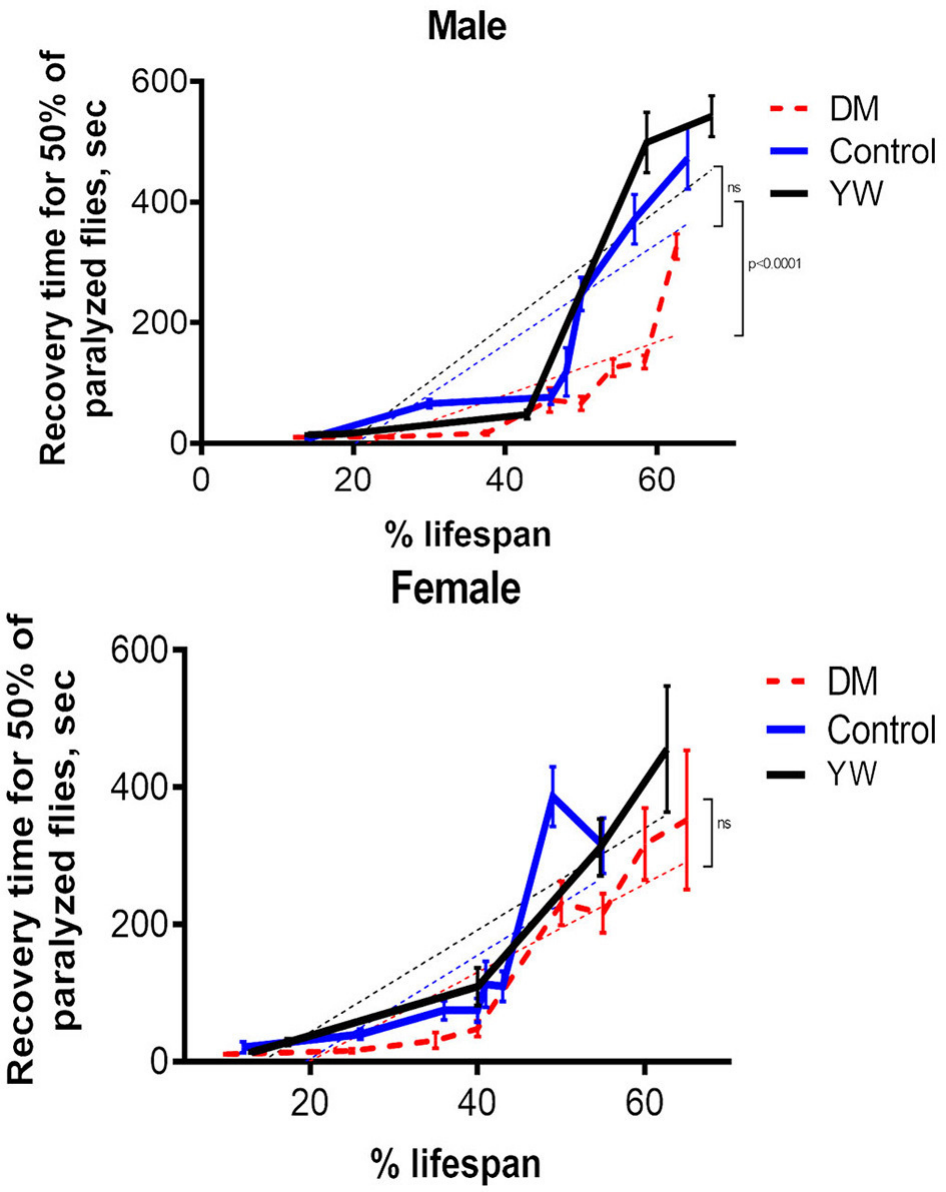
Acquired temperature sensitive paralysis. Measurements were done in the DM, control (Da-GAL4, *dprx5*/+), and *y w* flies at different ages (Supplementary Table 4). Results are means ± SEM normalized to percent of life span. The dotted lines represent linear regression analysis curves. There were no statistically significant differences in age-specific changes in recovery time for 50% of paralyzed female flies between the DM and control lines, as determined by analysis of the slopes of corresponding regression lines. Statistically significant differences were observed between the DM and control male flies (*P* < 0.0001). There were no significant differences between driver control (+/Da-GAL4*, dprx5*) and *y w* control.

It has been shown that temperature-sensitive paralysis, a marker of decreased neural transmission, is a biomarker of aging (Reenan and Rogina, 2008). As flies age they become progressively more susceptible to high temperatures displaying a paralysis phenotype that is presumably due to failure in neural signal transmission. Using a modified form of Reenan and Rogina's method, we observed this age-dependent increase in temperature sensitive paralysis in the driver and *y w* controls, and the DM flies (Figure 5). When time to recovery from paralysis at various time points in fly lifespan was scaled to percentage of lifespan, DM flies exhibited an increase in time to recovery that was comparable to control flies, suggesting that DM flies experience neuronal decline at a rate comparable to that of control flies when scaled with their shortened lifespan.

### Brain Morphology

We also investigated the brain morphology for vacuoles that normally indicate neurodegenerative processes (Kretzschmar et al., 2005) and found no significant difference between DM and single *prx3* and *prx5* mutants at different ages. The relatively low number of vacuoles (one-three) observed in the short-lived DM flies and a single *prx3* and *prx5* mutants that live almost normal life span (Supplementary Figure 1), would suggest that these signatures of neurodegeneration were probably not a causal factor for the mortality observed in the DM flies.

## Discussion

Consistent with previous studies (Radyuk et al., 2010; Odnokoz et al., 2016), we found that the DM flies develop characteristics normally associated with aging but at a significantly earlier age than control flies. In this study we found that the neuronal system is affected by global under-expression of mitochondrial Prxs. The double mutants exhibit disruption of sleep-wake behavior, impaired geo- and phototaxis, and failure in neuronal transmission (Figures 2–5). Some of these parameters, such as total activity and sleep were determined by physiological aging of DM flies while changes in other characteristics, such as onset of night sleep were more pronounced even in physiologically young flies, suggesting cumulative effects of aging and toxicity due to under-expression of mitochondrial Prxs.

Some of these parameters, such as changes in total activity and sleep, progressed throughout lifespan according to the physiological age of DM flies while changes in other characteristics, such as onset of the night sleep and temperature-sensitive paralysis were more pronounced even in physiologically young flies, suggesting cumulative effects of aging and toxicity due to under-expression of Mitochondrial Prxs.

Many studies showed that impairment of behavior in many species is a hallmark of aging and neurodegenerative disorders (Shaw et al., 2000; Koh et al., 2006; Froy, 2011; Umezaki et al., 2012). The changes in sleep-wake behavior during aging in *Drosophila* have been well-described (Martin and Grotewiel, 2006; Serway et al., 2009; Jones and Grotewiel, 2011; Koudounas et al., 2012; Ismail et al., 2015). In old age flies show decreased daily activity and increased total sleep. Their sleep becomes more fragmented, and the time between lights off and the end of an activity bout (the night activity offset), a circadian rhythmicity parameter, decreases (Koudounas et al., 2012). Studies on transgenic flies with altered expression of genes encoding antioxidants, showed accelerated age-dependent decline in locomotor behavior. For instance, both Sod1 and Sod2 RNAi flies displayed accelerated decline in the ability to climb and fly during aging (Oka et al., 2015). *trx-2* mutant flies also showed an accelerated age-dependent decline in climbing activity (Tsuda et al., 2010). Koh et al. showed that flies exposed to low concentration of paraquat throughout their life span showed an increase in sleep fragmentation (Cirelli, 2006; Koh et al., 2006). Our studies and those of others suggest that oxidative stress and damage, and compromised redox in the DM can contribute to deterioration of sleep-wake behavior in these flies. Since the nervous and muscular system are responsible for these behaviors, the age-related behavior changes observed in response to under-expression of mitochondrial Prxs are likely to be signs of deterioration of one or possibly both of these two key systems.

Another parameter that was altered in the DM was locomotor activity, which is known to progressively decline in both flies and humans during age and also impaired in neurodegenerative diseases (Ostchega et al., 2000; Jones and Grotewiel, 2011). There are also well-documented links between oxidative stress and changes in behavior (Sakashita et al., 2010; Truong et al., 2015). Fly strains that have increased resistance to oxidative stress displayed delayed impairment in locomotor behavior (Arking and Wells, 1990; Kang et al., 2002), and over-expression of enzymes, which protect from oxidative damage, showed positive effects on fly locomotor behavior (Chavous et al., 2001; Ruan et al., 2002). In contrast, under-expression of antioxidants, such as Sod1 and Sod2, display an accelerated decline in locomotor ability (Martin et al., 2009). Thus, the observed changes in locomotor activity in the DM can be attributed to OS/changes in redox found in the DM.

*Drosophila* aging is also associated with increased acquired temperature sensitive paralysis (Reenan and Rogina, 2008), which has also been induced experimentally in young flies through targeted mutations in ion channels, in synaptic transmission proteins and in other genes that result in lower levels of Na+ channels (Vijayakrishnan and Broadie, 2006). These data suggest that aging is associated with a decline in the same proteins. Our experiments with temperature sensitive paralysis in mitochondrial Prxs mutants demonstrate a similar rate of acquired temperature sensitive paralysis, scaled across their shortened lifespan (Figure 5), suggesting that under-expression of mitochondrial Prxs may potentially affect Na+ channels and cause dysregulation of synaptic signal transmission. Interestingly, another study conducted by our laboratory in flies with enhanced pro-reducing capacity found changes in transcription of the genes that control ion transport (Radyuk et al., 2012).

In attempts to identify a particular tissue responsible for the life-shortening phenotype, we investigated the effects of under-expression of mitochondrial Prxs with different neuronal drivers. Despite the fact that these drivers share some overlap in driving the expression of the target genes (Legan et al., 2008), D42 is more effective in the expression of target genes in motor neurons while APPL driver is characterized by high-level expression of the target genes in the brain.

Previously, we found that global under-expression of dPrx3 and dPrx5 has a variety of effects on organ systems, although no pro-apoptotic changes were observed in the brain tissue of the DM (Radyuk et al., 2010) or the control flies undergoing normal aging (Zheng et al., 2005), and no obvious signs of neurodegeneration of the brain tissue due to vacuolization (Supplementary Figure 1). However, there were still indications that neuronal function is affected in the DM, including acquired temperature sensitive paralysis (Figure 5), an indicator of failure in neuronal transmission that can be associated with motor-neuronal pathology. Early onset in disruption of sleep-wake behavior (Figures 3, 4) also suggests the effects on neurons that regulate motor activity and circadian behavior.

The effects of Prx under-expression in motor neurons, comparable to those observed in flies with global under-expression of mitochondrial Prxs, suggest that changes in redox specifically in this tissue can be a causal factor for rapid death and neurogenerative pathology. The *Drosophila* motor neurons are part of the giant fiber system, which mediates rapid escape behavior and has been used in studying behavior modifications such as seizures and paralysis (Pavlidis and Tanouye, 1995). The motor neurons innervate target muscles, such as indirect muscles and tergotrochantal muscles that are responsible for flying and jumping, respectively (Bravo-Ambrosio and Kaprielian, 2011). Since motor neurons transmit signals from CNS to target muscles, the changes in motor neurons caused by depletion of both Prxs may drive many of the age-related changes in behavior. Indeed, the deficit in climbing, movements toward a light source (Figure 2) and changes in multiple sleep-wake characteristics (Figure 4) may be due to a deterioration of motor neuron-connected muscles.

Previous studies have shown that in a wide range of species, the pathogenesis of many age-related diseases and mortality rate is associated with deterioration of muscles (Metter et al., 2002; Nair, 2005; Ruiz et al., 2008; Augustin and Partridge, 2009), suggesting the importance of normal skeletal muscle function in modulating systemic aging. The disruption in behavior and severe shortening of life span by removal of mitochondrial Prxs specifically in the motor neurons supports the common belief that maintenance of muscular function has a beneficial effect on organismal longevity (Chen et al., 2005; Boyle et al., 2009; Demontis and Perrimon, 2010).

Parkes et al. suggested that oxidative stress specifically in the motor neurons could be a critical causative factor in aging (Parkes et al., 1998). Besides, it is well-established that mutations in the gene encoding SOD1, an antioxidant enzyme catalyzing conversion of superoxide anion into hydrogen peroxide, are associated with loss of motor neurons in the spinal cord and in the brain (Rosen et al., 1993; Andersen, 2006). The failure of motor neurons is a causal factor in the development of ALS, the neurodegenerative disease affecting selectively both upper and lower motor neurons with consequent muscle atrophy, paralysis and eventually rapid death due to respiratory failure (Rosen et al., 1993; Robberecht and Philips, 2013; Chai and Pennetta, 2015). Experiments with the ALS model in *Drosophila* have shown that alleles with mutated SOD1 live shorter and are also characterized by change in redox, mainly in the ratio of GSH/GSSG (Mockett et al., 2003). It may be concluded that the motor neurons are very sensitive to changes in redox and are very sensitive to oxidative impairment, which is well-documented in both vertebrates and invertebrates (Simpson et al., 2003; Ferraiuolo et al., 2011; Smith et al., 2017).

Although we did not explore the effects of the DM specifically in the muscles, the data suggest that deterioration of thoracic muscles might also contribute to the shorter-lived phenotype. The muscles, which are under control of motor neurons, are rich in mitochondria and characterized by high metabolic rate. Since, mechanical, thermal, and oxidative stressors occur during muscle contraction (Arndt et al., 2010), a muscular tissue is particularly susceptible to damage compared to other tissues. Different stressors, such as paraquat and hypoxia, resulted in increased levels of apoptotic cells in the thorax, where motor neurons and muscles are located (Zheng et al., 2005). The removal of mitochondrial Prxs using the global driver also induced a strong apoptotic response in the thorax (Radyuk et al., 2010). Other studies have shown that changes in resistance to oxidative stress in muscle tissue can modulate life span (Vrailas-Mortimer et al., 2011). Thus, the increase in levels of mitochondrial SOD2 in *Drosophila* muscles delays age-related muscle dysfunction and extends life span (Vrailas-Mortimer et al., 2011). Future studies to investigate the role of mitochondrial Prxs in muscles are well-warranted.

The results of this and previous studies add new insights into the redox hypothesis of aging, specifically that dysregulation of redox signaling in a limited number of critical cell types may have a strong impact on longevity. This study provides evidence that redox changes in the motor neurons play a particularly important role in modulating longevity and determining the onset of age-dependent changes. It also suggests a critical role of redox balance in motor neurons in development of various pathologies. In this light, the changes in locomotor activity and dysregulation of sleep-wake behavior observed in the DM phenotype could signal a failure of neuromuscular control implicating neuronal and/or muscular dysfunction.

Surprisingly, pan-neuronal under-expression of mitochondrial Prxs resulted in little or no deficits in longevity or physical activity (Figures 1, 2, 4). Brain tissue is relatively deficient in antioxidant enzymes, rich in oxidizable substrates such as polyunsaturated fatty acids and catecholamines, and has a high level of ROS production (Chong et al., 2005; Lin and Beal, 2006). It is characterized by a higher rate of metabolism and lower capacity for regeneration as compared to other organs (Andersen, 2004) and therefore is highly susceptible to oxidative damage. Thus, this was unexpected, as studies conducted in our lab have shown that bolstered activity of other redox-affecting enzymes, GCLc and G6PD in the brain tissue had strong beneficial effects on life span while under-expression conferred the opposite effects (Orr et al., 2005; Legan et al., 2008). Over-expression of another Prx, the ER-localized dPrx4 in neuronal tissue also led to extension of life span (Klichko et al., 2016).

Overall the findings suggest that the impact on sleep-wake behavior is more likely due to motor neuron failure rather than brain degeneration. Why does depletion of dPrx3 and dPrx5 have such a minor effect on brain tissue? One possible reason is that sufficient dPrx3 suppression in critical tissues was not achieved with the APPL driver and there can be differences in tissue and cell specificity as well as efficiency between APPL and D42 drivers. Alternatively other Prxs may compensate for the depletion of Prx3 and Prx5 in this tissue. Among six distinct Prxs, *Drosophila* Jafrac1 is an ortholog and functional homolog of human PrxII (Lee et al., 2009). The neuronal over-expression of Jafrac1 prolonged, while the knockdown of Jafrac1 shortened, the *Drosophila* life span and affected mitochondrial function (Lee et al., 2009). Thus, Jafrac1 may play an important role in compensating the mitochondrial dysfunction found in flies under-expressing mitochondrial Prx3 and Prx5 (Radyuk et al., 2010; Odnokoz et al., 2016).

Another question that remains to be resolved is whether the under-expression of mitochondrial peroxiredoxins interferes with the neuronal transmission directly or acts indirectly through other signaling pathways that are affected by impaired mitochondrial function in these mutants. As was found in our previous studies using the DM transcriptome analysis, under-expression of mitochondrial Prxs influenced many biological processes and signaling pathways (Odnokoz et al., 2016). For example, it affects the immune pathways, where the state of the immune system changes to overactive / pro-inflammatory. It also influences sodium ion transport, which is known to be related to neuronal function. It also leads to oxidative damage. Thus, it remains to be determined which particular process/pathway is influenced by under-expression of Prxs in motor neurons.

To conclude, our studies confirm the essential role of mitochondrial Prxs in maintaining normal physiology and in preventing an early onset of mortality. We showed that the molecular, cellular and phenotypic hallmarks of normal physiological aging are generally observed in the DM flies and these changes scale with life span. We also found that changes in neuronal function and aging followed the same trend in males and females, suggesting that mitochondrial Prxs target pathways common to both sexes. Thus, potential interventions to alleviate pathologies associated with physiological decline due to under-expression of mitochondrial Prxs would be applicable to both sexes. Another important finding is that under-expression of Prxs in motor neurons determines the short-lived phenotype of DM and is also responsible for changes in physiological parameters. It is likely that mitochondrial Prxs play a more important role in maintaining neuromuscular function than that of the brain tissue. Thus, motor neurons appear to be a culprit for the physiological decline in activity and lifespan observed in the DM.

## Data Availability Statement

The raw data supporting the conclusions of this article will be made available by the authors, without undue reservation.

## Author Contributions

OO and SR: conceived and designed the experiments, performed the experiments, analyzed and interpreted the data, and wrote the paper. KN and DK: conceived and designed the experiments, performed the experiments, and analyzed and interpreted the data. CW and JC: performed the experiments and analyzed the data. VK and WO: analyzed and interpreted the data and contributed to writing the paper. All authors contributed to the article and approved the submitted version.

## Conflict of Interest

The authors declare that the research was conducted in the absence of any commercial or financial relationships that could be construed as a potential conflict of interest.
