# Supplementary material for: Mitochondrial Redox Signaling Is Critical to the Normal Functioning of the Neuronal System
Source: Front Cell Dev Biol. 2021 Jan 28;9:613036. doi: 10.3389/fcell.2021.613036 (PMC7876342; doi:10.3389/fcell.2021.613036)
Supplement: Supplementary file 1 [file Table_1.DOCX]

Supplementary Material

**Table S1. Ages of flies collected for the measurements of phototaxis and negative geotaxis.** Genotypes of fly lines are shown in Table 1. Asterisks indicate ages, which were used for linear regression analysis presented in Table 3.

| **MALES** | | | **FEMALES** | | |
| --- | --- | --- | --- | --- | --- |
| **Line** | **Age,**  **days** | **% of Life Span** | **Line** | **Age, days** | **% of Life Span** |
| **Appl**  **Control** | 29-30  49-51  50-51 | 39-40*  66-68*  67-68* | **Appl**  **Control** | 12-13  13-15  55-56  58-60 | 15-16*  16-19*  71-72*  75-77* |
| **Appl DM** | 28-29  34-37  47-49  55-56 | 39-40*  47-51*  65-68*  76-78 | **Appl DM** | 9-11  52-54  57-58 | 15-18*  85-88*  93-95 |
| **D42**  **Control** | 11-13  16-17  46-48  61-62 | 14-16  20-21  57-60*  76-77* | **D42**  **Control** | 9-10  12-13  49-50  59-60 | 11-13  14-15*  58-60*  70-71 |
| **D42 DM** | 9-13  13-17 | 42-60*  60-79* | **D42 DM** | 7-9  20-22  22-24 | 18-23*  50-55*  55-60* |

Table S2. Ages of flies collected for the sleep-wake behavior study depicted in Fig. 3. Genotypes of fly lines are shown in Table 1.

| **Line** | **Age,**  **days** | **% of Life Span** |
| --- | --- | --- |
| **Da Control** | 11-13  21-23  30-32  38-39  46-48 | 15  30  40  50  65 |
| ***dprx3*** | 10-11  20-22  29-31  35-37  45-46 | 15  30  40  50  65 |
| ***dprx5*** | 10-11  21-22  29-30  36-37  45-47 | 15  30  40  50  65 |
| **DM** | 3  6  8  10  13 | 15  30  40  50  65 |

Table S3. Ages of flies collected for the single fly sleep-wake behavior study. Ages of flies collected for the single fly sleep-wake behavior study depicted in Figure 4. Genotypes of fly lines are shown in Table 1. Asterisks indicate % of life span, which were used for linear regression analysis represented in Table 4.

| **MALES** | | |
| --- | --- | --- |
| **Line** | **Age** | **% of life span** |
| **Appl Control** | 29-30  50  50-51 | 39-40*  67*  67-68* |
| **Appl DM** | 28-29  34-35  48-49  55-56 | 39-40*  47-49*  67-68*  76-78 |
| **D42 Control** | 13  16-17  47  61-62 | 16  21  59*  77* |
| **D42 DM** | 10  14  16  18 | 47*  65*  77*  86* |

**Table S4. Ages of flies collected for the acquired temperature-sensitive paralysis experiments.** Da Control – *+*/Da-GAL4; *yw* – *yw* control; DM – double mutant.

| **MALES** | | | **FEMALES** | | |
| --- | --- | --- | --- | --- | --- |
| **Line** | **Age,**  **days** | **% of Life Span** | **Line** | **Age, days** | **% of Life Span** |
| **Da**  **Control** | 10  22  33  34  36  41  46 | 14  30  46  48  50  57  64 | **Da**  **Control** | 10  22  30  33  34  36  41  46 | 12  26  36  40  41  43  49  55 |
| ***yw*** | 10  13  30  41  47 | 14  19  43  59  67 | ***yw*** | 10  13  30  41  47 | 13  17  40  55  63 |
| **DM** | 3  6  9  11  12  13  14  15 | 13  25  38  46  50  54  58  63 | **DM** | 2  5  7  8  10  11  12  13 | 10  25  35  40  50  55  60  65 |

**Figure S1.** ***Drosophila* brain morphology.** Representative images showing vacuolization in paraffin sections from fly heads. One-three vacuoles were observed in 15 day old *dprx5* mutant (approximately 15-20 % of life span) and the DM (approximately 65% of life span), as well as 25 day old single *dprx3* and *dprx5* mutants (approximately 30-40% life span).


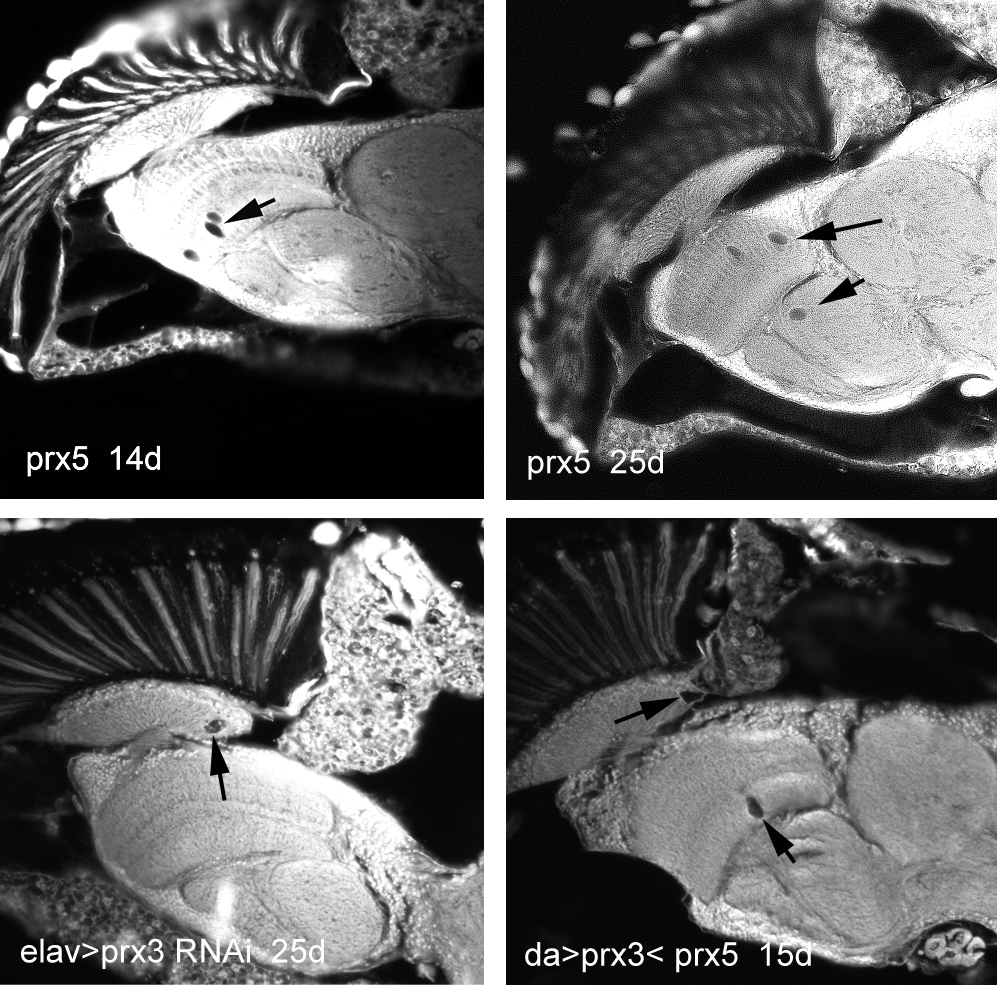


**Tissue sections and measurement of vacuolar pathology:** Newly eclosed flies, collected within 24h of eclosion, were aged for the appropriate time with the food vials exchanged with fresh ones every 4-5 days. Paraffin sections were prepared and analyzed for vacuole formation as described in (Sunderhaus and Kretzschmar, 2016). Briefly, whole flies were fixed in Carnoy’s solution and dehydrated in an ethanol series followed by incubation in methyl benzoate before embedding in paraffin. Sections were cut at 7μm and analyzed with a Zeiss Axioscope 2 microscope using the auto-fluorescence caused by the dispersed eye pigment.

Sunderhaus, E.R., and Kretzschmar, D. (2016). Mass Histology to Quantify Neurodegeneration in Drosophila. *J Vis Exp* (118). doi: 10.3791/54809.

**
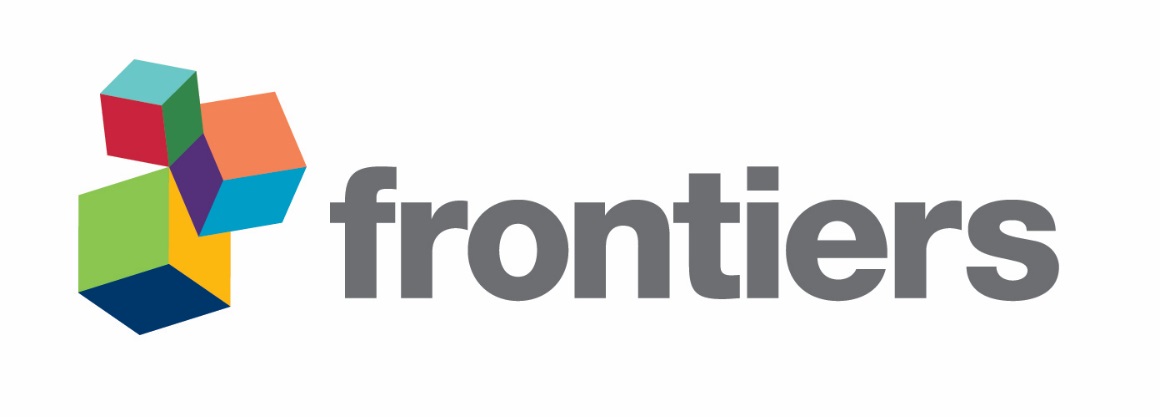
**
